# McCune-Albright Syndrome Mimicking Malignancy: an Endocrine Disease From Oncologist’s Perspective

**DOI:** 10.4274/Jcrpe.712

**Published:** 2012-09-11

**Authors:** D. Bahar Genç, M. Alp Özkan, Atilla Büyükgebiz

**Affiliations:** 1 Anadolu Medical Center, Department of Pediatric Oncology, Kocaeli, Turkey; 2 İstanbul Bilim University, Department of Pediatric Endocrinology, İstanbul, Turkey

**Keywords:** Fibrous dysplasia, malignancy, McCune-Albright syndrome

## Abstract

Fibrous dysplasia (FD) is categorized as either monostotic or polyostotic and may occur as a component of McCune-Albright syndrome (MAS). Imaging findings can mimic neoplastic diseases. We present a case of MAS initially suspected to have neoplastic disease. A 9-year-old girl was admitted to pediatric emergency with ataxia. Upon hospitalization, an extradural mass was seen on cranial magnetic resonance imaging (MRI) and the bone survey showed lytic lesions in the long bones. The patient was referred to the pediatric oncology department with a presumptive diagnosis of Langerhans cell histiocytosis or metastatic tumor. Further investigations demonstrated that the patient had MAS and coexisting postinfectious cerebellitis. The findings in this patient demonstrate that the radiographic findings and the clinical presentation of FD and MAS may be similar to those of malignant diseases.

**Conflict of interest:**None declared.

## INTRODUCTION

Fibrous dysplasia (FD) is a common benign disorder of the bone in which fibro-osseous tissue replaces the bony spongiosa. It comprises around 7-10% of all benign bone tumors. 70-80% of FD cases are monostotic and 20-30% are polyostotic (1). McCune-Albright syndrome (MAS) is defined by the triad of FD, café au lait spots, and precocious puberty or at least one of the typical hyperfunctioning endocrinopathies in almost any possible combination (2). Bone lesions observed in MAS and FD can mimic malignant processes (3). Besides, coexisting neoplasia is a rare but probable occurrence and malignant transformation develops in 0.5-4% of the cases of FD/MAS (4). Computerized tomography (CT) and conventional radiographic findings demonstrate the characteristics of FD. However, magnetic resonance imaging (MRI) and FDG (fludeoxyglucose)-PET-CT imaging appearances of FD often resemble those of tumors. This potential diagnostic pitfall could lead to unnecessary medical interventions (5,6,7). 

We report the case of a patient who had multifocal bone lesions and ataxia and was referred to our clinic with a presumptive diagnosis of malignancy. The patient was subsequently diagnosed as MAS and postinfectious cerebellitis. 

## CASE REPORT

A 9-year-old girl was admitted to the pediatric emergency department because of a 2-day-history of unsteady gait, vomiting, and dizziness. She was the third child of four pregnancies to a non-consanguineous couple. Her prenatal/natal history was unremarkable and her two siblings were healthy. One sibling had died in utero at the 8^th^ month of pregnancy without an identified pathology. The patient’s history did not reveal any recent immunization, upper respiratory tract infection, diarrhea or drug use. On inspection, the patient appeared to be in a state of exhaustion. Her blood pressure was 110/70 mm Hg, respiratory rate 24/minute and pulse rate 80/minute. Her height was 135 cm (50-75^th^ perc., SDS: 0.9) and weight was 27 kg (25-50^th^ perc.). Her body mass index (BMI) was 14.8. Physical examination findings were within normal limits except for ataxia. There were two irregularly bordered café au lait spots - one on the right cheek and the other in the presacral area. Breast development and pubic hair growth both corresponded to Tanner stage 4. 

**Figure 1:**
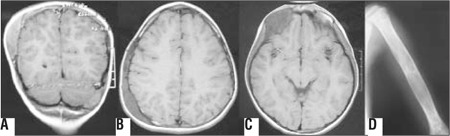
**A:** Coronal magnetic resonance imaging (MRI) showing extradural mass lesion at parietal area, **B:** Axial MRI showing fusiform expansion of skull bones extending through occipital area, **C:** MRI demonstrating frontal mass, **D:** Plain film of humerus revealing lytic lesions with ground glass appearance

Initial laboratory findings were within normal ranges. A presumptive diagnosis of acute postinfectious cerebellitis was made. Parenteral hydration and acyclovir therapy were initiated. Otolaryngology consultation was sought. An inner ear problem as cause of ataxia was considered, but could not be proven by audiological examination. Cranial MR was done to delineate central nervous pathologies and revealed that there were fusiform expansions reaching 1.5 cm in thickness in almost all skull bones with a special predominance in the occipital and right parietal bones. Moderate diffuse contrast enhancement was also noted in these bones. Brain parenchyma was normal in terms of white and grey matter. The radiological differential diagnosis included Langerhans cell histiocytosis, primary bone lymphoma and neuroblastoma metastasis. CT scans supported the MRI findings except for a ground glass appearance indicative of FD (Figure 1). 

The bone survey findings were as follows: loss of the distinction between cortex and medulla at the cranium, fingers and toes, as well as the humeri, radii, and ulnae, in addition to disrupted trabecular structure and presence of radiolucent areas. ^99m^Tc MDP scintigraphy demonstrated extensive multiple osteoblastic uptake in accordance with either histiocytosis or FD. 

The lytic and expansive bone lesions raised the suspicion of malignancy, and the patient was referred to the department of pediatric oncology. Re-evaluation of the patient's history revealed that the breast development and pubic hair growth had started 2^6/12^ years before the current situation. Precocious puberty, café au lait spots, and polyostotic FD in radiological differential diagnosis pointed to MAS. However, due to a suspicion of malignancy and presence of coexisting ataxia, a bone marrow biopsy was performed to exclude malignant infiltration. The biopsy represented both cortical and medullary areas. There were no signs of neoplasia, but the trabeculae were thin and mimicked Chinese letters, lacking osteoblastic rimming and were surrounded by a moderately cellular fibroblastic proliferation. The findings were consistent with FD. 

The patient was therefore referred to the department of pediatric endocrinology. The patient’s bone age was 11 years. Thyrotropin (TSH) was found to be slightly elevated (5.07 mU/mL). Serum alkaline phosphatase level was 738 U/L, Ca 10 mg/dL, and phosphorus 5 mg/dL. Her DEXA results revealed a Z-score (lumbar spine) of -1.9. The patient was diagnosed as a case of MAS. Her symptoms disappeared after 10 days of acyclovir treatment and supportive measures. She was discharged to be followed up jointly by the orthopedics and pediatric endocrinology departments. The patient had menarche at age of 9^8/12^ years.

## DISCUSSION

FD is a developmental anomaly of the bone formation in which the normal bone and marrow are replaced by fibrous tissue and small, woven spicules of bone. FD may exist in a monostotic or polyostotic form. MAS is characterized by a combination of FD, cutaneous pigmentation, and endocrine abnormalities (2). The mutations in the regulatory Gsα protein - G protein α subunit - (encoded by the [i]GNAS[/i] gene) are the underlying molecular etiology of MAS. The disease is not inherited as MAS results from postzygotic somatic mutation (8). The earlier the mutation occurs in embryogenesis, the more widespread are the tissues involved. Mutations late in embryogenesis are more focused and account for mild cases, with only one or two of the three manifestations of the syndrome which makes the diagnosis more difficult (9). 

The diagnosis of MAS is usually established on clinical grounds. The medical history including extraskeletal manifestations should be taken into consideration. The initial evaluation of our patient failed to reveal her precocious puberty and therefore MAS was not among the differential diagnoses considered for the multifocal skeletal involvement. The diagnosis became evident when the concomitant presence of polyostotic FD, café au lait spots, and precocious puberty was evaluated. Unfortunately, [i]GNAS[/i]mutation testing was not available in our facility. 

The detection of a bone mass with/without limp or fracture poses a diagnostic dilemma for the general pediatrician or orthopedic surgeon in deciding whether the mass is cancerous or indicates FD. A radiograph is usually the first imaging modality to be performed. The plain film reveals a lytic, ground glass lesion in FD. In case of doubt about the diagnosis, the CT is helpful in demonstrating the medullary-based, fibro-osseous nature of FD (1). After the diagnosis of FD, bone scintigraphy or a skeletal survey should be done to determine the extent of the disease. However, that is where the second question comes in: Whether the patient has isolated FD or MAS with atypical or partial form. The classical triad of MAS includes precocious puberty, café au lait spots, and FD. This classical definition, however, does not hold for patients with partial and atypical forms of the syndrome (8, 10). A single endocrinopathy other than precocious puberty, such as isolated Cushing’s syndrome in the neonatal period, might be the first manifestation of MAS (11,12). Fortunately, endocrine manifestations serve to increase the awareness of the physician and lead to a more detailed diagnostic assessment. In the absence of other signs of the syndrome, MAS diagnosis may be delayed until the development of classical findings. Hannon et al (13) reported that the pediatric FD cases were not routinely being evaluated for MAS. In this study, 5 of the 9 children with FD were found to have café au lait spots and 3 to have thyroid dysfunction. [i]GNAS[/i] mutations were positive in 5 of the 9 cases. The authors concluded that a diagnosis of MAS might be overlooked in a substantial proportion of children with FD of the bone. In a study from France, the authors searched for the activating [i]GNAS[/i] mutations in 113 patients presenting with different signs of MAS. Three of 7 patients with isolated FD were positive for [i]GNAS[/i] mutations (8). In a meta-analysis done by Lee et al (14), 9 studies searching [i]GNAS[/i] mutations in 203 cases of sporadic FD were analyzed. The overall positivity rate for [i]GNA[/i]S mutation was 71.9% (146/203). These studies draw attention to the likelihood of MAS in cases with isolated FD at initial presentation.

After diagnosis, MAS patients are referred to pediatric endocrinology clinics. However, the clinical dilemma may still persist in MAS cases presenting with mass lesions. [i]GNAS[/i] mutations responsible for MAS lead to adenylyl cyclase-mediated increase in cAMP which initiates uncontrolled cell proliferation (15). Thus, FD possesses the potential for malignant change. Although very rare, its frequency was estimated to be 0.5% for monostotic FD and 4% for MAS (4). Several reports have documented the presence of thyroid, breast, pituitary and adrenocortical tumors in patients with MAS (16). In addition, activating mutations of [i]GNAS[/i] have been identified in adrenal hyperplasia, ovarian cysts, thyroid carcinomas, adrenocortical, pituitary, renal and Leydig cell tumors (17). Furthermore, coexisting FD and malignancy in independent sites can cause potential diagnostic confusion in terms of metastatic disease (3,18,19). 

Some of the MAS cases may present with atypical initial features such as ovarian masses and testicular enlargement. In an otherwise healthy child who presents with a pelvic/testicular mass, the distinction between malignant and benign lesions can be challenging. Such patients may undergo unnecessary diagnostic or therapeutic interventions. Nabhan et al (6) reported that 4 out of 9 patients presenting with ovarian masses underwent unnecessary salpingo-oophorectomy and were all later diagnosed with MAS and benign ovarian cysts. In a study from Macedonia, a 3-year-old girl with a mass at the left ovary had surgery and the pathology of the salpingo-oophorectomy revealed a benign ovarian cyst (5). In Khanna et al.’s (7) and Arrigo et al.’s (20) studies, two boys presenting with unilateral macroorchidism underwent testicular biopsy in order to exclude malignancy. Ovarian cysts that can be encountered in MAS cases may also require invasive measures like laparotomy (12,21) (Table 1). In a European Collaborative Study, 113 patients who showed at least one of the signs of MAS were investigated with regard to activating Gsα mutations. Of the tissues analyzed, 13, 11 and 1 of the samples belonged to ovary, bone and testis, respectively. The ovarian tissue samples were reported to be obtained from ovariectomy. The reason for tissue sampling was not given. However, since only 27 patients out of 113 MAS cases in this series displayed the complete classical triad, it can be hypothesized that the invasive tissue sampling was done in an effort to identify the underlying pathology before the MAS was diagnosed (8). Since the radiographic findings and clinical presentation of FD and MAS may be similar to those of malignant diseases, and also considering the overlooked MAS cases with isolated FD followed in orthopedics clinics, it would be highly probable to increase the number of cases undergoing tissue sampling for suspected malignancy. 

**Conflict of Interest**

The findings in this patient demonstrate that the radiographic findings and the clinical presentation of FD and MAS may be similar to those of malignant diseases.

## Figures and Tables

**Table 1:**
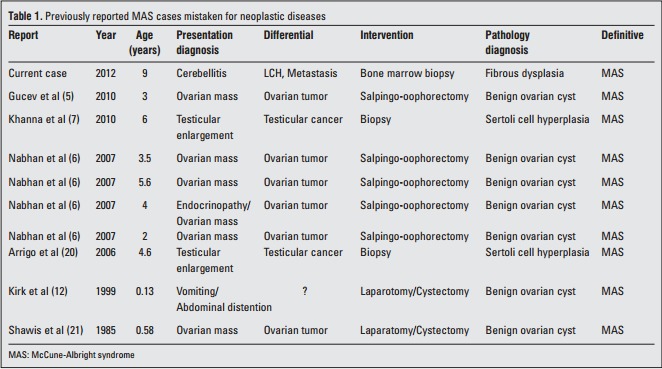
Previously reported MAS cases mistaken for neoplastic diseases
